# Wernicke Encephalopathy Related to Hyperemesis Gravidarum: A Retrospective Study of 12 Cases

**DOI:** 10.1155/crcc/7607058

**Published:** 2025-02-02

**Authors:** Kaouthar Abouelbaqua, Houssam Rebahi, Nisrine Louhab, Najib Kissani, Ahmed Rhassane El Adib

**Affiliations:** ^1^Anesthesia and Intensive Care Medicine Department, Mohammed VI University Hospital of Marrakech, Marrakech, Morocco; ^2^Laboratory of Childhood, Health & Development, Faculty of Medicine and Pharmacy of Marrakech, Cadi Ayyad University, Marrakech, Morocco; ^3^Neurology Department, Mohammed VI University Hospital of Marrakech, Marrakech, Morocco

**Keywords:** guidelines, hyperemesis gravidarum, magnetic resonance imaging, thiamin, Wernicke encephalopathy

## Abstract

**Objective:** Wernicke encephalopathy (WE) related to hyperemesis gravidarum (HG) is a devastating neuropsychiatric syndrome that remains frequently undiagnosed in pregnant women. Although many cases have been published, more studies are required to establish guidelines for the early detection and treatment of this condition.

**Material and Methods:** We conducted a retrospective study to analyze the available data concerning 12 cases of WE complicating HG in the Mother and Child Hospital's Obstetric Intensive Care Unit, belonging to Mohamed VI University Hospital of Marrakesh.

**Results:** Twelve out of 76 HG admitted cases developed WE. Pregnant WE patients became depleted after 11 weeks of vomiting at median gestational weeks of 16.2. They had a severe weight loss of more than 5% of their body and had all presented prodromal signs of WE before the actual onset of the clinical triad. WE diagnosis was clinically made based on Caine's operational criteria as they allow early identification of the disease. A good tool that could also aid diagnosis is an encephalic MRI; however, it should not delay treatment with prompt administration of high doses of thiamin of > 500 mg/day. Chronic sequelae in this study occurred in 45.5% and death in one case.

**Conclusion:** In HG, thiamin rapidly depletes which can lead to WE with adverse outcomes for the mother and fetus. Therefore, physicians must be vigilant in detecting early signs of WE to promptly provide a high dose of thiamin with targeted multimodal therapies as this could be lifesaving.

## 1. Introduction

Wernicke encephalopathy (WE) is an acute neuropsychiatric syndrome and a medical emergency characterized by a clinical triad of eye movement disorders, mental status change, and ataxia as a result of thiamin (vitamin B_1_) depletion. Although WE is mainly observed in alcoholic patients, who represent 50% of the total reported cases, it has been linked to other conditions such as anorexia nervosa, prolonged starvation, malnutrition, malignant and gastrointestinal disorders, chronic kidney disease, dialysis, thyrotoxicosis, organ transplantation, total parenteral nutrition (PN), and hyperemesis gravidarum (HG) [1]. HG is commonly defined as the occurrence of more than three episodes of vomiting per day, associated with a weight loss of more than 5% of body weight. This condition complicates up to 3% of pregnancies and leads to fluid, electrolytes, acid–base imbalance, nutrition deficiencies, acute kidney injury (AKI), and neurological involvement mainly dominated by WE [1, 2]. An early identification of the acute phase of the disease allows total reversibility of its symptoms and favorable maternal–fetal outcome. However, WE following HG is often misdiagnosed as the association of HG-WE is relatively unknown. We report our experience of WE pregnant women to review the clinical characteristics of this devastating disease and raise the clinician's index of suspicion of the diagnosis.

## 2. Methods

### 2.1. Study Design

This retrospective study depicts the available data concerning 12 cases of WE following HG in Mother and Child Hospital's Obstetric Intensive Care Unit (OICU) belonging to Mohammed VI Teaching Hospital of Marrakesh, within 6 years, between January 2016 and December 2021.

### 2.2. WE

The 12 cases were diagnosed with WE based on Caine's operational criteria, which require the presence of two or more of the following four major signs: (1) dietary deficiencies, (2) oculomotor abnormalities, (3) cerebellar dysfunction, and (4) either an altered mental state or memory impairment [3].

### 2.3. HG

HG diagnosis was based on common criteria cited in different guidelines including the onset, during the first trimester, of protracted and tenacious nausea and vomiting with a severe Motherisk Pregnancy-Unique Quantification of Emesis (PUQE-24) score (ranging from 13 to 15), reduction of oral intake and weight loss of more than 5%, dehydration, and electrolyte abnormalities, with no other etiologies that could explain this clinical presentation of nausea and vomiting in pregnancy (NVP) [2].

## 3. Results

During this period, 12 out of 76 HG cases developed WE. The mean maternal age was 30.5 (16–46) years old. Various sociodemographic characteristics were identified including urbanism (83.3% or 10/12), housewives (83.3% or 10/12), low education (75% or 9/12), poor income (75% or 9/12), multigravida (58.3% or 7/12), and history of hospital admission for HG in a previous pregnancy (16.6% or 2/12).

The median gestational weeks (GWs) of NVP appearance was 6.6 (6–12) GWs. The severity of vomiting was determined by the Motherisk PUQE-24 score. They had a severe weight loss of > 5% of their body weight. Net weight loss in kilogram was documented in 9/12 cases, and on average, they had lost 17 (8–32) kg. They had been vomiting for a median of 11 (6–12) weeks before the onset of the neurological symptoms at a median GW of 16.2 (12–26) GWs. However, one had presented WE symptoms on Day 6 after delivery (Table 1).

Prodromal signs of WE were present in all of the patients, namely, nausea, vomiting, loss of appetite, fatigue, and weakness in 100% (12/12). However, in pregnant women, the likelihood of missing these symptoms is higher as they fully overlap with HG symptoms. Additional prodromal characteristics of WE that were identified in our cases are difficulty in concentration in 33.3% (4/12); blurred vision, anxiety, and memory impairment in 16.6% (2/12); and finally, insomnia, apathy, and diplopia in 0.8% (1/12) (Table 1).

The most frequently observed feature from the classical triad was altered mental status. More specifically, confusion was present in all of the patients (12/12), followed by problems in alertness, cognition, and hallucinations in 33.3% (4/12); agitation and disorientation in 25% (3/12); and memory impairment in 16.6% (2/12), and 8.3% (1/12) showed signs of apathy. Oculomotor abnormalities affected 91% (11/12) of patients. The most frequent symptom was nystagmus detected in 91.6% (11/12) (Video S1), followed by ophthalmoplegia in 33.3% (4/12). Ataxia was found in 83% (10/12) (Table 2).

Additional associated neurological abnormalities were also identified, such as reduced muscle strength in the lower and upper limbs in 83.3% (10/12) and 33.3% (4/12), respectively; areflexia in 16.6% (2/12); paresthesia in 25% (3/12); and dysarthria in 41.6% (5/12) (Table 2).

Laboratory screening revealed anemia, with a low hematocrit level in 33.3% (4/12), hyponatremia and hypokalemia in 66.6% (8/12), raised urea and creatinine levels in 25% (3/12), elevated ALT and AST in 75% (9/12), and a low thyroid-stimulating hormone (TSH) level in 91.6% (11/12).

**Figure 1:**
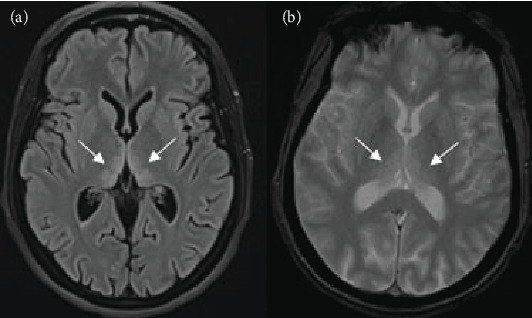
MRI showing hypersignal lesions (arrows) in the thalami on axial FLAIR (a) and T2-weighted (b) sequences.

**Figure 2:**
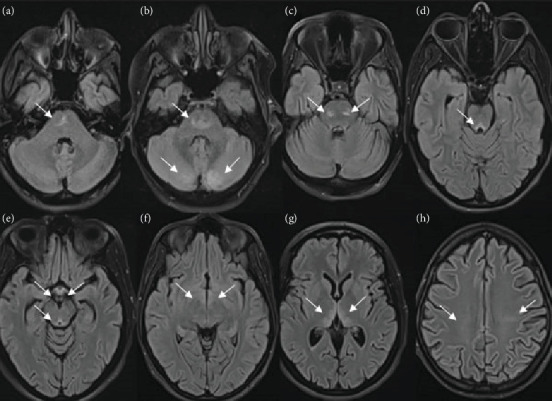
MRI of patients showing hypersignal lesions (arrows) on axial FLAIR in (a) the brainstem; (b) central pons and cerebellum; (c) red nuclei; (d) tectal plate; (e) mammillary bodies and periaqueductal region; (f) wall of the third ventricle; (g) thalami, paraventricular region, and frontal lobe; and (h) centrum semiovale.

Magnetic resonance imaging (MRI) scan was done in all patients and showed in 66.6% (8/12) of the cases various brain lesions consistent with WE. The median average interval between the symptoms' onset and the acquisition of images was 5.4 (1–23) days. In regard to the cases with positive MRI findings, typical encephalic involvement was identified as bilateral and symmetrical hypersignal in the following regions: The thalamic region was affected in 87.5% (7/8) (Figure 1), periaqueductal region mammillary bodies in 71.4% (5/7), and third ventricle in 37.5% (3/8), while atypical lesions were as follows: centrum semiovale, tectal plate, and the frontal lobe in 25% (2/8) and brainstem, corpus callosum, red nuclei, and cerebellum in 12.5% (1/8) (Figure 2). In addition, one patient had an increased signal in mid pons which was highly suggestive of central pontine myelinolysis association.

For the initial management of HG, our patients were put on different hydration protocols after admission, depending on their dehydration state and serum electrolyte level. An average volume of 2.2 L of either 5% dextrose or 0.9% saline or a combination of both was administered per 24 h. In addition, electrolyte supplementation including Na^+^, K^+^, and Ca^2+^ at an average of 55.4, 35.4, and 25.4 mmol, respectively, was added in each bag according to the patients' daily monitoring of serum electrolyte level.

Various antiemetics were prescribed, namely, pyridoxine in 91.6% (11/12), metopimazine in 33.3% (4/12), metoclopramide in 66.6% (8/12), and ondansetron in 50% (6/12). Moreover, an antiacid treatment and thromboprophylaxis were also used.

Thiamin was prescribed as a WE-specific treatment. A total of 66.6% (8/12) received an average of 311.8 mg/24 h via IV for 2 weeks, whereas 33.3% (4/12) received a mean dose of 1083 mg/24 h orally and maintained it for a longer period until birth.

The current study's mean hospitalization length was 22.8 (10–51) days. Regarding the pregnancy evolution, three different outcomes were recorded: 41.6% (5/12) of fetuses did not survive, 18.1% (2/12) of fetuses were born prematurely with a low Apgar score < 7, and 41.6% (5/12) of fetuses were born on term without any complication (Table 3).

The follow-up declared a total recovery in 33.3% (4/12) cases, whereas 50% (6/12) had persistent neurological symptoms, which were assessed based on the modified Rankin scale (Table 3), associated in 16.6% (2/12) with memory impairment and in 8.3% (1/12) with dysarthria. Death occurred in one case.

## 4. Discussion

Although in recent years, there has been an increase in the number of clinical settings in which WE is encountered, this fatal disease is still highly underdiagnosed as one autopsy study confirmed that WE was suspected in only about one-third of alcoholic and 6% of nonalcoholic patients during their lifetime. Alcohol abuse–related WE remains the most reported causative factor, with a prevalence of 12%–14% in the general population. Epidemiological data concerning WE related to HG on the other hand are very lacking—with an estimated prevalence of 0.04%–0.13% in the overall nonalcoholic population—it is believed that almost 80% of the cases remain undiagnosed during their lifetime [1, 4–6]. In this study, only WE patients with HG were included, with an estimated frequency of 14.4%. This high incidence could be explained by physicians' unawareness of WE diagnosis in HG patients.

### 4.1. Patients' Characteristics

Most studies agreed that HG is more common among young-aged mothers [2, 7, 8], especially in the presence of various sociodemographic characteristics that were commonly identified in the literature and this study, such as residency in urban regions, low educational level, being a housewife, and poor income [1, 7–11].

WE following HG usually occurs at 14–16 weeks of gestation, following more than 3 weeks of vomiting [12, 13]. Oudman et al. reported an average pregnancy duration of 15.3 weeks before WE is depicted after a median of 7 weeks of vomiting and a severe weight loss of > 12 kg in HG cases. This was a longer period compared to regular morning sickness in pregnancy and often continued until later in pregnancy. This finding was attributed to thiamin deficiency as a causative factor, especially in cases that develop vomiting after 10 weeks of gestation or for a prolonged time exceeding 4 weeks [14]. Excessive weight loss has been described as a major contributing factor to WE as well, as it is usually a sign of insufficient nutritional intake or nutrients being lost in the body, Therefore, physicians must be specifically vigilant in detecting and treating WE in patients with sudden and severe weight loss and vomiting as these two could be major warning signs and strong predictors of WE [15]. The average onset of WE in our cases was at 16 GWs; However, one of our patients had a late onset of the symptoms at Day 6 postpartum, which was not previously reported. In this study, the HG patients had a significant weight loss of more than 5% of their body weight, with an average of 17 kg documented in 9/12 cases. Although this may seem relatively high, a weight loss of over 12 kg is not uncommon in HG, and a massive weight loss prior to the development of Wernicke–Korsakoff syndrome (WKS) was identified as a profound characteristic of nonalcoholic WKS [15].

### 4.2. Prodromal Characteristics and Classic Triad

In this study, all patients developed early signs of thiamin deficiency before the actual onset of WE clinical features (Table 1). Similarly, all 177 HG cases reported by Oudman et al. had prodromal signs of WE. However, NVP and loss of appetite are very common during gestation, and the likelihood of missing these symptoms is higher as they fully overlap with HG symptoms [14, 15]. Additional common warning signs that have to draw physicians' attention are double and blurred vision [1, 14].

Retrospective studies have shown that altered consciousness is the most prevalent presenting symptom of the clinical triad (82%), followed by ocular signs (29%) and a variety of ataxia (23%) [4, 16, 17]. Nevertheless, In the HG population, behavioral and mental disturbances did not frequently appear according to numerous reviews, which could be explained by the young age of pregnant women, as it was identified as a protective factor against all forms of reactive mental status change [18]. Consequently, many authors highlight the importance of recognizing sensorimotor changes, such as diplopia and eye movement problems, specifically in young pregnant women with HG [1, 14]. Similar to what was reported, the most prevalent symptom in this study was altered consciousness. Ocular signs, on the other hand, were also predominant, as 91 6% of our cases had nystagmus (Video S1).

### 4.3. Caine et al. Criteria

WE is a clinical diagnosis classically characterized by the acute onset of the typical triad. However, with a sensitivity of only 23%, the majority of HG patients exhibit only one of these symptoms, while 19% may not have any of the signs [7, 13]. Caine et al. developed a proposed set of criteria with a sensitivity of 94% and specificity of 99% for WE diagnosis. These criteria were later adopted by the European Federation of Neurologic Societies (EFNS) as they allow the disease precocious diagnosis [3, 19].

In this study, 81.8% of cases had fulfilled all items of this clinical triad, while 18.8% had two clinical items combined with a context of dietary insufficiency caused by persistent NVP. Hence, all cases had met the Caine et al. criteria and were therefore diagnosed with WE.

### 4.4. Other Neurological Symptoms

A total of 83.3% of our patients had presented signs of peripheral neuropathy, such as paresthesia; decreased muscle strength; reduced power, especially in the lower limbs, with an inability to maintain Mingazzini; and diminished tendon reflexes. Studies have associated features of peripheral neuropathy, such as distal dominant sensory disturbances, decreased muscle strength, or decreased deep tendon reflexes in the extremities, with thiamin deficiency, complicating approximately 80% of the known cases of WKS and probably contributing to the ataxic gait. Acute polyneuropathy was reported in many HG cases as well. According to Di Gangi et al., 60.3% of the patients had features of peripheral neuropathy [1, 12, 20–22].

Additional atypical manifestations of WE, including dysarthria, brisk reflexes, and hypertonia, were identified as well, which suggests the involvement of other areas of the brain, as multiple studies attributed these findings to the involvement of the upper motor neuron and pontine in WE. According to Chiossi et al., six out of 48 patients had developed signs suggesting central pontine or extrapontine myelinolysis, such as Babinski's sign, spastic quadriparesis, hyperreflexia, dysphagia, conjugate gaze palsy, spastic dysarthria, and facial paralysis [1, 7, 23, 24].

### 4.5. Imaging

MRI scan is currently considered the most valuable method to confirm the diagnosis, as well as to rule out other alternative diagnoses; however, with a high specificity of 93% and a low sensitivity of only 53%, the absence of neuroradiological findings does not exclude the disease. Additionally, the timing of the imaging study and the total dose of thiamin at the time of acquisition correlate with its sensitivity. Therefore, it should be ideally performed before thiamin administration, as brain abnormalities are quickly reversed after treatment, which can be challenging due to the urgent need for treatment at the acute stage of the disease [1, 25]. The median average interval between the symptoms' onset and the acquisition of images in our patients was 5.4 (1–23) days, and we believe that the significant delay in some cases may be due to the misdiagnosis of the disease in the HG population.

MRI sequences, including T2-weighted scan, fluid-attenuated inversion recovery (FLAIR), and diffusion-weighted imaging (DWI), typically show bilateral and symmetrical hypersignals in the paraventricular thalamic regions, the hypothalamus, the mammillary bodies, the periaqueductal region, and the floor of the fourth ventricle. However, these “typical” lesions are usually found in only 58% of WE cases. Unusual sites include the putamen, caudate nucleus, splenium of the corpus callosum, dorsal medulla, pons, red nucleus, substantia nigra of the midbrain, cranial nerve nucleus, vermis, and dentate. These atypical lesions are more frequently found in nonalcoholic WE [1, 4, 19, 26]. According to Oudman et al., 68.9% or 122/177 HG cases underwent MRI. In 91% or 111/122 of those cases, this exam demonstrated the presence of radiological alterations in the thalamic region, corroborating the WE diagnosis [1, 14]. Likewise, the most predominant lesion in our patients is thalamic injuries found in 87.5% (7/8) of cases with positive MRI findings.

### 4.6. Laboratory Findings

Numerous laboratory disturbances found in this study were reported in the literature as well such as anemia [12, 27], low hematocrit level due to hemoconcentration in the setting of dehydration [3], prerenal failure due to volume depletion [6, 28], hyponatremia and hypokalemia due to severe hyperemesis [28], and elevated liver enzymes as patients with cytolysis were reported to be more likely to develop WE than those with normal values [23, 29]. Thiamin depletion was also more likely to be aggravated by preexisting hyperthyroidism or gestational thyrotoxicosis [1, 2, 13, 23, 28].

In patients with suspected WE, determining blood thiamin concentrations or measuring the red blood cell transketolase activity could be a useful confirmatory test for thiamin deficiency, especially for cases with ambiguous presentations. However, these tests lack specificity and sensitivity; they are unavailable in most clinical laboratories, and treatment cannot be delayed to wait for the results [1]. In this study, thiamin measurement was not included in the patients' laboratory investigations due to their unavailability in our hospital and the urgency of rapid thiamin administration in the early stages of the disease.

### 4.7. Management

The cornerstone of WE treatment remains the timely administration of thiamin. However, there is a lack of consensus regarding its adequate dosage. The EFNS guidelines recommend an intravenous infusion of 200 mg of thiamin diluted with 100 mL of normal saline or 5% dextrose, given over 30 min, three times daily until clinical improvement stops [19]. British authors, on the other hand, have recommended 500 mg given during a 15–30-min interval in a mixture of saline solution or dextrose, three times per day, for 2–3 days, followed by 250 mg daily until improvements cease [30]. Moreover, the systematic review by Oudman et al. suggests a positive correlation between higher treatments (> 500 mg) and faster and better outcomes [14]. In this study, thiamin was administered via the IV route in only 66.6% of the patients, and the dosage was below the recommendations, whereas, in 33.3%, the vitamin was administered orally, which could interfere with its serum concentration, and that was essentially due to the unavailability of the intravenous form of thiamin in our country. More favorable outcome was noted in the patients who had received higher dosages of the vitamin, especially via the IV route.

Other adjuvant therapies should be prescribed in HG cases, such as antiacid medications and antiemetics (Table 4), as they have been associated with PUQE-24 scores lowering and life quality improvement [2, 27, 31]. Nutrition support, on the other hand, should be centralized around fluid resuscitation and electrolytes' stabilization, followed by nutritional repletion to support adequate fetal growth. Determining which fluid regimen is most appropriate could be challenging as there is no evidence to determine which is most appropriate. However, given that most women with HG present with hyponatremia, hypochloremia, and hypokalemia and are ketotic, it seems appropriate to use normal saline with additional potassium chloride in each bag. Physicians should consider the degree of dehydration and must be guided by daily monitoring of electrolytes, especially in the setting of severe hyponatremia (it should not be corrected faster than 10 mmol/L per 24 h to prevent central pontine myelinolysis). Magnesium and phosphorus depletion is another significant factor that should be considered as severe deficiency may lead to a refractory response to thiamin, and that is due to its role as a major cofactor in the catalytic action of many enzymes implicated in the conversion of thiamin into thiamine pyrophosphate [1, 32]. Intravenous sugar solutions should be avoided as the disease may be precipitated by carbohydrate-rich food and IV dextrose. However, in case of minimal oral intake, starvation, or uncontrolled nausea, dextrose administration could be considered after adequate correction of thiamin deficiency [1, 31].

Once nausea and vomiting have stopped and electrolytes are stable, more advanced nutrition support (ANS) therapies such as enteral nutrition (EN) and PN should be started in those with severe symptoms. In the setting of HG, it is impossible to have a single nutrient deficiency but rather an inadequate intake of glucose, protein, vitamins, and minerals as there are 37 nutrients in the Recommended Dietary Allowances (RDAs) some of which could present similar to thiamin deficiency and should be explored [32]. Numerous predictive equations with varying complexity are currently available to estimate energy needs in pregnancy. While some account for age, height, weight, and activity level, others are simple weight-based equations, such as the equation of resting energy expenditure (REE) during pregnancy in kilocalories: REE = 346.43943 + 13.962564 × *W* + 2.700416 × *H* − 6.826376 × *A* (*W*, weight; *H*, height; and *A*, age) [33]. Physicians should assume that all HG patients are at risk of refeeding syndrome; therefore, EN and PN should be initiated at no more than 50% of the estimated total calorie intake and slowly advanced by 33% every 1–2 days. Once oral intake meets 75% of the estimated calorie, protein, and fluid needs, ANS can be discontinued. Monitoring the response to EN and PN should include a close assessment of malnutrition, refeeding risk, tolerance to interventions, glycemic control, maternal weight gain, and fetal growth [32, 34].

The combination of pregnancy, immobility, and dehydration is likely to increase the risk of thrombosis in women admitted with HG. Therefore, thromboprophylaxis should be prescribed alongside adequate hydration, mobilization when possible, and thromboembolic stockings. The Royal College of Obstetricians and Gynecologists guidelines suggest using low-molecular-weight heparin in all admitted women with HG unless there are specific contraindications [1, 35].

### 4.8. Evolution

Studies have shown that HG patients are more likely to be hospitalized and bedridden for a prolonged time than other nonalcoholic WE cases [15]. Although the initial improvements in acute symptoms can be observed within the first week, it usually takes 1–3 months to resolve. On follow-up, residual nystagmus and ataxia are seen in 60% of patients, while chronic memory disorders are seen in up to 80% as WE patients may progress to Korsakoff syndrome. Maternal mortality rate ranges from 20% to 30%, whereas the fetal mortality rate can go as high as 50% [1, 4, 5, 7, 17, 28]. In this study, residual neurological disorders were found in 45.5% of the cases and were associated with chronic memory impairment in 18%. Meanwhile, total resolution of symptoms was noted in 33.3% of the patients who had received the IV format of the vitamin at a dosage higher than 1000 mg/day. Fetal mortality is estimated at 41.6% and is highly likely due to severe maternal weight loss and prolonged malnutrition [32]. Maternal death in this study occurred in one case (Table 3).

In summary, in HG, thiamin rapidly depletes, which can lead to WE with adverse fetomaternal outcomes. Therefore, physicians should be aware of this serious but preventable disease (WE) by its early diagnosis to promptly provide a high dose of thiamin with targeted multimodal therapies and aggressive medical nutrition therapy as this could be lifesaving. The proposed guidelines (Table 4) provide applicable assessment methods to both prevent and detect WE related to HG and a management plan to treat this misdiagnosed disorder and even avoid its occurrence in pregnant women.

## Figures and Tables

**Table 1:** Onset and prodromal signs of Wernicke encephalopathy.

**Case number**	**Onset of vomiting**	**Onset of neurological symptoms**	**Total weight loss**	**Signs of thiamin deficiency**
Case 1	9 GWs	16 GWs	25 kg	Loss of appetite, nausea, vomiting, anxiety, and difficulty in concentration
Case 2	4 GWs	24 GWs	—	Loss of appetite, nausea, vomiting, fatigue, and weakness
Case 3	7 GWs	13 GWs	10 kg	Loss of appetite, nausea, vomiting, fatigue, weakness, anxiety, and memory loss
Case 4	5 GWs	16 GWs	15 kg	Loss of appetite, nausea, vomiting, fatigue, and weakness
Case 5	2 GWs	13 GWs	17 kg	Loss of appetite, nausea, vomiting, fatigue, weakness, and apathy
Case 6	10 GWs	16 GWs	11 kg	Loss of appetite, nausea, vomiting, fatigue weakness, blurred vision, diplopia, insomnia, and difficulty in concentration
Case 7	6 GWs	16 GWs	8 kg	Loss of appetite, nausea, vomiting, fatigue, weakness, and difficulty in concentration
Case 8	2 GWs	12 GWs	20 kg	Loss of appetite, nausea, vomiting, fatigue, and weakness
Case 9	8 GWs	26 GWs	—	Loss of appetite, nausea, vomiting, fatigue, and weakness
Case 10	12 GWs	Day 6 postpartum	15 kg	Loss of appetite, nausea, vomiting, fatigue, weakness, and difficulty in concentration
Case 11	9 GWs	13 GWs	32 kg	Loss of appetite, nausea, vomiting, fatigue, weakness, difficulty in concentration, and memory loss
Case 12	6 GWs	14 GWs	—	Loss of appetite, nausea, vomiting, fatigue, weakness, and blurred vision

Abbreviation: GWs: gestational weeks.

**Table 2:** Clinical features of WE in HG patients.

	**WE triad**			**Dysarthria**	**Signs of peripheral neuropathy**
	**Mental status changes**	**Eye movement disorders**	**Gait and trunk ataxia**		
Case 1	Confusion, disorientation, problems in alertness and cognition, memory impairment	Nystagmus	Present	Absent	Present
Case 2	Confusion	Nystagmus, ophthalmoplegia	Present	Absent	Present
Case 3	Confusion, memory impairment	Nystagmus	Present	Absent	Present
Case 4	Confusion, disorientation, agitation, problems in alertness and cognition	Nystagmus, ophthalmoplegia	Present	Absent	Present
Case 5	Hallucinations, apathy	Absent	Absent	Absent	Present
Case 6	Confusion	Nystagmus	Present	Present	Present
Case 7	Confusion, disorientation, agitation, hallucinations, problems in alertness and cognition	Nystagmus	Present	Present	Present
Case 8	Confusion, agitation	Nystagmus	Present	Present	Absent
Case 9	Confusion	Nystagmus	Absent	Absent	Present
Case 10	Confusion, problems in alertness and cognition	Nystagmus, ophthalmoplegia	Present	Absent	Absent
Case 11	Confusion, hallucinations	Nystagmus	Present	Present	Present
Case 12	Confusion, hallucination	Nystagmus, ophthalmoplegia	Present	Absent	Absent

**Table 3:** Follow-up of WE-HG patients.

	**Pregnancy outcome**	**RS at discharge**	**Period before the follow-up**	**RS at the follow-up**	**Other persistent neurological symptoms**
Case 1	Fetal loss	3	3 months	2	Memory impairment
Case 2	Fetal loss	6			
Case 3	Healthy baby on term	3	6 months	1	No other associated symptoms
Case 4	Healthy baby on term	4	9 months	2	Memory impairment
Case 5	Healthy baby on term	4	1 month	3	No other associated symptoms
Case 6	Premature birth with low Apgar score < 7	5	1 year and 4 months	4	No other associated symptoms
Case 7	Fetal loss	3	1 year and 8 months	1	No other associated symptoms
Case 8	Fetal loss	5	1 year and 7 months	4	Dysarthria
Case 9	Fetal loss	2	1 month	1	No other associated symptoms
Case 10	Premature birth with low Apgar score < 7	3	1 month	1	No other associated symptoms
Case 11	Healthy baby on term	5	1 month	4	No other associated symptoms
Case 12	Healthy baby on term	4	2 weeks	3	No other associated symptoms

*Note:* 0, no symptoms; 1, no significant disability: able to carry out all usual activities, despite some symptoms; 2, slight disability: able to look after own affairs without assistance, but unable to carry out all previous activities; 3, moderate disability: requires some help, but can walk unassisted; 4, moderately severe disability: unable to attend to own bodily needs without assistance and unable to walk unassisted; 5, severe disability: requires constant nursing care and attention, bedridden, and incontinent; 6, dead.

**Table 4:** Proposed guidelines for diagnosis, prevention, and management of WE related to HG.

Initial management of HG	
Diagnosis	• Severe NVP (Motherisk PUQE‐24 > 13) • Onset in the first trimester • Reduction of oral intake and weight loss of more than 5% of body weight • Dehydration and electrolyte abnormalities • No other causes of vomiting
Investigations	FBC, urea and creatinine, electrolytes, LFTs, *β*hcg, and TSH (if required) Obstetric ultrasound
General recommendations	Diet as tolerated Cease iron; maintain folate and iodine
Medications	Ondansetron 4–8 mg PO/IV BD-TDS Consider dosing with either • Metoclopramide 10 mg PO/IV • Chlorpromazine 10–25 mg PO/IV • Dimenhydrinate 25–50 mg PO/IV • Promethazine 25 mg PO/IV Consider prednisone 40–50 mg OD, or hydrocortisone 100 mg IV BD, or dexamethasone 8 mg OD Wean prednisone over 7–10 days (may be continued until symptoms resolve)
Adjuvant therapy	• IV fluids 1–3× per day as required + electrolytes in each bag depending on serum electrolyte level • PPI: esomeprazole or omeprazole 20 mg PO/IV OD-BD • Thromboprophylaxis: LMWH, thromboembolic stockings, and mobilization
WE prophylaxis	Thiamin: 100–200 mg IV/IM: before any carbohydrate administration and if poor oral intake, especially in the presence of early thiamin deficiency signs (nausea, vomiting, loss of appetite, blurred/double vision, difficulty in concentration, memory loss, anxiety, apathy, insomnia, fatigue, and weakness)
WE management	
Diagnosis	Requires two of the following four signs: • Dietary deficiencies • Oculomotor abnormalities: nystagmus and ophthalmoplegia • Cerebellar dysfunction: gait and truck ataxia • Altered mental state and/or memory impairment
Investigations	• MRI (should not delay treatment) • Blood thiamin measurement (if available)
Treatment	Thiamin 500 mg IV TDS for 3 days • In case of absence of clinical response, thiamin should be stopped • In responders, 250 mg of IV/IM thiamin should be administered daily for the next 5 days or until clinical improvement stops
Prophylaxis	Thiamin > 10 mg/day PO is recommended until delivery (may be maintained for a longer period in breast-feeding mothers)

Abbreviations: BD: twice a day; *β*hcg: human chorionic gonadotropin; FBC: full blood count; IM: intramuscular; IV: intravenous; LFTs: liver full tests; LMWH: low-molecular-weight heparin; OD: once a day; PO: per os; TDS: three times a day; TSH: thyroid-stimulating hormone.

## Data Availability

The data used to support the findings of this study are available on request from the corresponding author, Kaouthar Abouelbaqua.

## Supporting Information

Supporting Information Additional supporting information can be found online in the Supporting Information section. Video S1: https://drive.google.com/file/d/1A7__Vgz9QVBrxha2oOENyltowWrGSEUW/view?usp=share_link. Nystagmus in one of our admitted cases. A 40-year-old pregnant woman who developed HG at a gestational age of 9 weeks. She had a severe Motherisk PUQE-24 score (> 13) and had been vomiting for 7 weeks approximately before the onset of WE complete clinical triad. MRI revealed radiological injuries in the following sites (arrows): thalami, mammillary bodies, and periaqueductal region.

## References

[1] Abouelbaqua K. (2022). *Wernicke Encephalopathy Following Hyperemesis Gravidarum: Obstetric Intensive Care Unit Experience, [Ph.D. thesis]*.

[2] Austin K., Wilson K., Saha S. (2019). Hyperemesis gravidarum. *Nutrition in Clinical Practice*.

[3] Caine D., Halliday G. M., Kril J. J., Harper C. G. (1997). Operational criteria for the classification of chronic alcoholics: identification of Wernicke’s encephalopathy. *Journal of Neurology, Neurosurgery, and Psychiatry*.

[4] Sechi G., Serra A. (2007). Wernicke’s encephalopathy: new clinical settings and recent advances in diagnosis and management. *The Lancet Neurology*.

[5] Donnino M. W., Vega J., Miller J., Walsh M. (2007). Myths and misconceptions of Wernicke’s encephalopathy: what every emergency physician should know. *Annals of Emergency Medicine*.

[6] Kantor S., Prakash S., Chandwani J., Gokhale A., Sarma K., Albahrani M. J. (2014). Wernicke’s encephalopathy following hyperemesis gravidarum. *Indian Journal of Critical Care Medicine: Peer-reviewed, Official Publication of Indian Society of Critical Care Medicine*.

[7] Salahuddin N. K., Dutta A., Qureshi N. A. (2019). Hyperemesis gravidarum, Wernicke encephalopathy, and Korsakoff syndrome: a critical appraisal of the relevant literature. *Journal of Advances in Medicine and Medical Research*.

[8] Gabra A. (2018). Risk factors of hyperemesis gravidarum: review article. *Health Science Journal*.

[9] Nurmi M., Rautava P., Gissler M., Vahlberg T., Polo-Kantola P. (2020). Incidence and risk factors of hyperemesis gravidarum: a national register-based study in Finland, 2005-2017. *Acta Obstetricia et Gynecologica Scandinavica*.

[10] Fiaschi L., Nelson-Piercy C., Tata L. J. (2016). Hospital admission for hyperemesis gravidarum: a nationwide study of occurrence, reoccurrence and risk factors among 8.2 million pregnancies. *Human Reproduction*.

[11] Ioannidou P., Papanikolaou D., Mikos T., Mastorakos G., Goulis D. G. (2019). Predictive factors of hyperemesis gravidarum: a systematic review. *European Journal of Obstetrics & Gynecology and Reproductive Biology*.

[12] Chiossi G., Neri I., Cavazzuti M., Basso G., Facchinetti F. (2006). Hyperemesis gravidarum complicated by Wernicke encephalopathy: background, case report, and review of the literature. *Obstetrical & Gynecological Survey*.

[13] Ismail S. K., Kenny L. (2007). Review on hyperemesis gravidarum. *Best Practice & Research Clinical Gastroenterology*.

[14] Oudman E., Wijnia J. W., Oey M., van Dam M., Painter R. C., Postma A. (2019). Wernicke’s encephalopathy in hyperemesis gravidarum: A systematic review. *European Journal of Obstetrics & Gynecology and Reproductive Biology*.

[15] Oudman E., Wijnia J. W., Oey M. J., van Dam M., Postma A. (2021). Wernicke-Korsakoff syndrome despite no alcohol abuse: a summary of systematic reports. *Journal of the Neurological Sciences*.

[16] Harper C. G., Giles M., Finlay-Jones R. (1986). Clinical signs in the Wernicke-Korsakoff complex: a retrospective analysis of 131 cases diagnosed at necropsy. *Journal of Neurology, Neurosurgery & Psychiatry*.

[17] Flynn A., Macaluso M., Inna D., Troutman M. M. (2015). Wernicke’s encephalopathy. *The Primary Care Companion For CNS Disorders*.

[18] Patti L., Gupta M. (2021). Change in Mental Status. *StatPearls*.

[19] Galvin R., Bråthen G., Ivashynka A. (2010). EFNS guidelines for diagnosis, therapy and prevention of Wernicke encephalopathy. *European Journal of Neurology*.

[20] Ishibashi S., Yokota T., Shiojiri T. (2003). Reversible acute axonal polyneuropathy associated with Wernicke-Korsakoff syndrome: impaired physiological nerve conduction due to thiamine deficiency?. *Journal of Neurology, Neurosurgery & Psychiatry*.

[21] Huertas-González N., Hernando-Requejo V., Luciano-García Z., Cervera-Rodilla J. L. (2015). Wernicke’s encephalopathy, wet beriberi, and polyneuropathy in a patient with folate and thiamine deficiency related to gastric phytobezoar. *Case Reports in Neurological Medicine*.

[22] Liang H., Wu L., Liu L. L., Han J., Zhu J., Jin T. (2017). A case report: non-alcoholic Wernicke encephalopathy associated with polyneuropathy. *Journal of International Medical Research*.

[23] Kotha V. K., De Souza A. (2013). Wernicke’s encephalopathy following hyperemesis gravidarum: a report of three cases. *The Neuroradiology Journal*.

[24] Divya M. B., Kubera N. S., Jha N., Jha A. K., Thabah M. M. (2022). Atypical neurological manifestations in Wernicke’s encephalopathy due to hyperemesis gravidarum. *Nutritional Neuroscience*.

[25] Silva A., Almeida-Xavier S., Lopes M., Soares-Fernandes J., Sousa F., Varanda S. (2023). Is there a time window for MRI in Wernicke encephalopathy—a decade of experience from a tertiary hospital. *Neurological Sciences*.

[26] Manzo G., De Gennaro A., Cozzolino A., Serino A., Fenza G., Manto A. (2014). MR imaging findings in alcoholic and nonalcoholic acute Wernicke’s encephalopathy: a review. *Biomed Research International*.

[27] Lowe S. A., Armstrong G., Beech A. (2020). SOMANZ position paper on the management of nausea and vomiting in pregnancy and hyperemesis gravidarum. *The Australian & New Zealand Journal of Obstetrics & Gynaecology*.

[28] Di Gangi S., Gizzo S., Patrelli T. S., Saccardi C., D’Antona D., Nardelli G. B. (2012). Wernicke’s encephalopathy complicating hyperemesis gravidarum: from the background to the present. *The Journal of Maternal-Fetal & Neonatal Medicine*.

[29] Smith T. J., Johnson C. R., Koshy R. (2021). Thiamine deficiency disorders: a clinical perspective. *Annals of the New York Academy of Sciences*.

[30] Braillon A. (2021). Alcohol withdrawal syndrome in neurocritical care unit: nicotine replacement therapy and thiamine deficiency. *Neurocritical Care*.

[31] Campbell K., Rowe H., Azzam H., Lane C. A. (2016). The management of nausea and vomiting of pregnancy. *Journal of Obstetrics and Gynaecology Canada*.

[32] Erick M. (2022). Gestational malnutrition, hyperemesis gravidarum, and Wernicke’s encephalopathy: what is missing?. *Nutrition in Clinical Practice*.

[33] Hronek M., Zadak Z., Hrnciarikova D., Hyspler R., Ticha A. (2009). New equation for the prediction of resting energy expenditure during pregnancy. *Nutrition*.

[34] Elkins J. R., Oxentenko A. S., Nguyen L. A. B. (2022). Hyperemesis gravidarum and nutritional support. *The American Journal of Gastroenterology*.

[35] *The Management of Nausea and Vomiting of Pregnancy and Hyperemesis Gravidarum (Green-top Guideline No. 69)*.

