# Supplementary material for: Wernicke Encephalopathy Related to Hyperemesis Gravidarum: A Retrospective Study of 12 Cases
Source: Case Rep Crit Care. 2025 Feb 2;2025:7607058. doi: 10.1155/crcc/7607058 (PMC11824303; doi:10.1155/crcc/7607058)
Supplement: Supporting Information — Additional supporting information can be found online in the Supporting Information section. Video S1: https://drive.google.com/file/d/1A7__Vgz9QVBrxha2oOENyltowWrGSEUW/view?usp=share_link. Nystagmus in one of our admitted cases. A 40-year-old pregnant woman who developed HG at a gestational age of 9 weeks. She had a severe Motherisk PUQE-24 score (> 13) and had been vomiting for 7 weeks approximately before the onset of WE complete clinical triad. MRI revealed radiological injuries in the following sites (arrows): thalami, mammillary bodies, and periaqueductal region. [file 7607058.f1.pdf]

Supplementary material

Video 1

https://drive.google.com/file/d/1A7__Vgz9QVBrxha2oOENyltowWrGSEUW/view?usp=share_link

Nystagmus in one of our admitted cases. A 40-year-old pregnant woman who developed HG at a gestational age of 9 weeks. She had a severe Motherisk PUQE-24 score (>13) and had been vomiting for 7 weeks approximately before the onset of WE complete clinical triad. MRI revealed radiological injuries in the following sites (Arrows): thalami, mammillary bodies and periaqueductal region.
